# Decellularized Extracellular Matrices for Skin Wound Treatment

**DOI:** 10.3390/ma18122752

**Published:** 2025-06-12

**Authors:** Rui Liang, Ruliang Pan, Li He, Yu Dai, Yuting Jiang, Shujun He, Baoguo Li, Yuli Li

**Affiliations:** 1 Shaanxi Key Laboratory for Animal Conservation, College of Life Science, Northwest University, Xi’an 710069, China; liangrui620@stumail.nwu.edu.cn (R.L.); ruliang.pan@nwu.edu.cn (R.P.); 18872055081@163.com (L.H.); daiyu@nwu.edu.cn (Y.D.); jyt@nwu.edu.cn (Y.J.); baoguoli@nwu.edu.cn (B.L.); 2 International Centre of Biodiversity and Primate Conservation, Dali University, Dali 671003, China; 3 School of Human Science, The University of Western Australia, Perth, WA 6009, Australia; 4 Shaanxi Institute of Zoology, Xi’an 710032, China; shujunhe91@163.com; 5 College of Life Science, Yanan University, Yanan 716000, China

**Keywords:** decellularized matrix, extracellular matrix, skin trauma, wound healing, biomaterials

## Abstract

Skin trauma, especially chronic trauma, poses a significant clinical challenge, often leading to severe disability or even death. Traditional treatment methods exhibit several limitations in terms of efficacy, material availability, and biocompatibility. The development of decellularized extracellular matrices (dECMs) has led to revolutionary progress in this field. These materials retain the bioactive components of the natural extracellular matrix (ECM) and, combined with their excellent physical structure, promote wound healing. Preclinical studies have demonstrated that dECM-based dressings can enhance the re-epithelialization rate by 20–50% and shorten the healing cycle of chronic wounds by 40%. This article systematically reviews the application of dECM in wound repair. First, it outlines the pathophysiology of wound healing, focusing on the mechanisms by which key ECM components promote wound healing. Next, it classifies decellularized materials and proposes material design schemes for different types of damage. Finally, the limitations of current dECM-based wound treatments and future research directions are discussed. This review aims to provide a theoretical framework and technical reference for researchers in related fields, promoting the widespread application of dECM materials for skin trauma treatment.

## 1. Introduction

The skin serves as a barrier against complex external environments, playing a crucial protective role [1]. However, its fragile nature makes it susceptible to various forms of damage [2]. Although the human body possesses intrinsic multicellular self-regulatory mechanisms for wound healing, this process can be disrupted by various factors [3]. Bacterial infections, excessive inflammation, and inadequate wound care may lead to chronic wound infections, which are commonly observed in populations with diabetes and cardiovascular diseases, as well as among the elderly [4,5,6]. Untreated chronic wounds may result in amputation, sepsis, or even death [7]. Additionally, in cases of severe trauma, significant scarring may occur alongside loss of skin function, imposing substantial psychological and social burdens on patients [8]. Therefore, effective wound management represents a critical clinical issue [9].

Traditional strategies for treating skin wounds primarily focus on providing passive protection during the healing process. However, existing skin substitute materials face various limitations, such as low survival rates, challenges in precisely replicating the complex physiological structure of natural skin, and limited capacity for promoting tissue regeneration [10]. While synthetic biomaterials such as hydrogels offer controllable mechanical properties and degradation rates through chemical modifications, their homogeneous composition lacks the dynamic bioactive signals of the natural extracellular matrix (ECM). Consequently, these materials cannot actively guide functional regeneration, limiting their clinical translation [11]. In contrast, decellularized extracellular matrix (dECM) demonstrates unique advantages in skin repair. Derived from natural tissues through decellularization techniques, dECM removes potentially immunogenic components (e.g., cellular debris and DNA) while preserving core ECM constituents such as collagen, glycosaminoglycans, and growth factors [12]. Its three-dimensional topological structure closely mimics the physiological microenvironment, providing natural adhesion sites and dynamic biological signals that promote cell migration, differentiation, and angiogenesis [13]. Nevertheless, the composition and mechanical properties of dECM remain challenging to precisely control [14]. Therefore, integrating the manufacturing precision of synthetic materials with the biologically favorable characteristics of dECM could enable more effective tissue remodeling [11].

Various decellularization methods have been developed for preparing dECM, including physical (e.g., repeated freezing and thawing, pressurization), chemical (e.g., acids, bases, detergents), and enzyme-mediated biological (e.g., trypsin, nucleases) approaches [15,16]. These methods aim to remove immunogenic cellular components from native tissues while preserving extracellular biomolecules and other bioactive molecules in the ECM [17,18]. Although chemical agents play a key role in the decellularization and removal of immunogenic material from decellularized tissues, they may damage the ECM and degrade biocompatibility [19,20]. Physical techniques are less toxic but exhibit low decellularization effectiveness, and they are thus typically used as adjunctive therapies that enhance the overall efficacy of decellularization [19]. Enzymatic decellularization, through targeted hydrolysis by trypsin/nuclease in combination with chemical detergents, can achieve > 95% removal of cellular and nuclear content [21,22]. However, enzyme residues may trigger an immune response, necessitating a thorough wash cycle. After decellularization, the absence of cells and nuclei should be verified through hematoxylin and eosin (H&E) and 4′,6-diamidino-2-phenylindole (DAPI) staining. Quantitative measurement of residual DNA content should show that the double-stranded DNA does not exceed 50 ng per mg of dry weight ECM and that the fragment length does not exceed 200 base pairs [23]. To assess the residual protein content of the ECM, additional characterizations (such as colorimetric assays, sodium dodecyl sulfate–polyacrylamide gel electrophoresis, and histological analysis) should be conducted, assessing structural proteins such as collagen, fibronectin, and laminin, as well as glycosaminoglycans (GAGs) and growth factors (GFs) [24].

In the field of skin regeneration, research on the use of dECM has achieved groundbreaking progress. Studies have shown that skin-derived dECM, prepared through optimized decellularization techniques, not only retains key components of the basement membrane but also uses its three-dimensional (3D) topological structure to induce the polarized migration and directional differentiation of keratinocytes [25]. To meet the diverse clinical needs of wound repair, researchers have successfully integrated advanced technologies such as hydrogels, 3D bioprinting, and electrospinning for the functional reprocessing of dECM. For instance, ECM-derived hydrogels extracted from decellularized porcine small intestinal submucosa have been demonstrated to support cell growth and viability, while promoting the expansion of endoderm-derived human organs in culture [26]. Additionally, decellularized ECM has been used as bioink in various biomedical applications. Kim et al. used skin-derived extracellular matrix bioink to 3D print prevascularized skin patches, which promoted in vivo wound healing [27]. Furthermore, studies have shown that decellularized ECM can be combined with electrospinning technology for enhanced wound healing [28]. At present, the application of dECM biomaterials has expanded beyond skin regeneration, advancing the engineering of organs, including the heart [29], kidney [13], liver [30], and lung [31]. Notably, the exceptional pro-angiogenic capacity of these materials plays a critical role in tissue regeneration.

This review focuses on dECM for cutaneous wound therapy, summarizing recent advances in the field. First, the wound-healing process is introduced, and the roles of key ECM components in wound repair are described. Subsequently, an overview of commonly used decellularized biomaterials and their applications in wound repair research is provided. The application of dECM to various types of wounds, including burn, diabetic, and infected wounds, is summarized. Finally, challenges in and potential future research directions for dECM-based trauma therapy are discussed. This review aims to enhance the understanding of dECM among researchers in related fields and stimulate its widespread application in skin trauma treatment.

## 2. Skin Wound Healing

**Figure 1:**
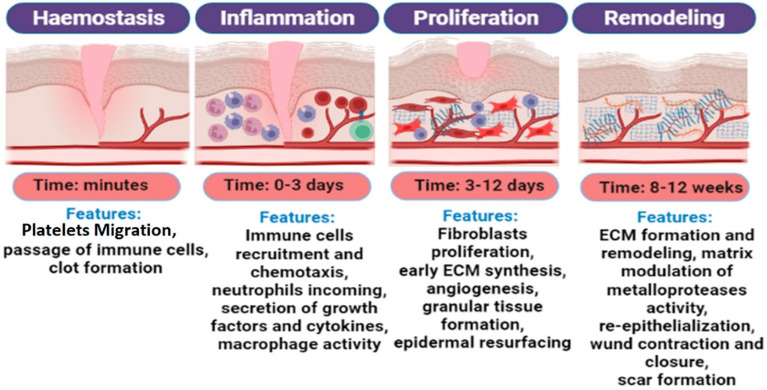
The different stages of the wound-healing process and their characteristics. Reprinted with permission from Ref. [40]. Copyright 2022 MDPI.

Skin wound healing is a complex and dynamic physiological process involving the regeneration of the epithelium and dermis, often resulting in scar formation. This process requires precisely coordinating various cell types and their secreted molecules to restore damaged tissues [32]. The typical wound-healing process is divided into four overlapping phases based on cellular events and biochemical reactions: hemostasis, inflammation, proliferation, and remodeling (Figure 1). These four phases are essential for proper wound healing [33,34].

Following injury, ruptured blood vessels expose subendothelial collagen to platelets. This exposure triggers a series of activations and accumulations in the coagulation cascade reaction [35], which then initiates the hemostatic process, the initial stage of wound healing. Blood-clot formation seals the damaged blood vessels. At the same time, phagocytes are recruited to the wound site to remove debris and microorganisms [36], initiating the inflammatory phase. Cytokines released by various cells act as chemoattractants, directing white blood cells to the wound site, where macrophages play a central role in inflammation [37,38]. The proliferative phase is characterized by re-epithelialization, angiogenesis, and the proliferation of fibroblasts. During this phase, fibroblasts synthesize new ECM components to rebuild the tissue structure, while endothelial cells promote angiogenesis [39]. Finally, the remodeling phase involves wound maturation and further re-epithelialization, with specialized cell types promoting complete wound healing and tissue recovery.

Although wound healing is typically a well-orchestrated and tightly regulated series of events, various factors can influence its outcome [33]. Oxidative stress, hormonal disturbances, infections, and other undesirable external factors (e.g., cigarette smoking, substance abuse, and environmental exposure) can all negatively affect wound healing [9]. These factors can induce chronic inflammation, leading to delayed healing and, in some cases, the formation of granulomas and abscesses. dECM-based biomaterials offer therapeutic potential at nearly every stage of wound repair.

## 3. Effect of Extracellular Matrix Components on Skin Wound Healing

The ECM is a noncellular, protein-rich matrix that provides structural support and cell attachment [41,42]. It ensures the integrity of tissues and organs and facilitates multidirectional communication among cells by imparting structural support and providing bioactive GFs and cytokines [43,44,45]. The ECM directly regulates multiple aspects of cellular behavior, including adhesion, proliferation, migration, and survival [46,47]. Indirect regulation associated with the ECM involves cellular actions on the ECM that stimulate extracellular protease secretion and regulate growth-factor bioavailability [32]. The cutaneous ECM primarily consists of collagen, elastin (EL), laminin (LM), fibronectin (FN), GAGs, and proteoglycans, along with molecules involved in ECM turnover, such as matrix metalloproteinases (MMPs) and thrombospondin-1 (TSP-1) [48]. Table 1 outlines the primary components and their corresponding percentages in skin ECM. Understanding the specific roles of these key ECM components in wound healing provides a scientific foundation and inspires new strategies for wound treatment.

### 3.1. Role of Collagen in Wound Healing

Collagen is a significant component of the skin ECM [53], providing tensile strength [54]. Several collagen types, primarily Types I, III, V, VII, XVII, and XVIII, play crucial roles in skin wound healing. Collagen Type I induces keratinocyte migration, initiates re-epithelialization, enhances the expression of matrix metalloproteinases in keratinocytes, and promotes matrix remodeling [33]. Additionally, collagen Type I exhibits immunomodulatory properties. Karin demonstrated that structures enriched with Type I collagen induce immunosuppressive functions in M2 macrophages and may effectively treat chronic diabetic wounds [55]. Type I and III collagens are rich in structural domains that bind vascular hemophilic factors and play a key role in regulating bone morphogenetic protein activity and promoting angiogenesis [56]. Collagen V is mainly involved in regulating fibroblast proliferation and migration [56]. Collagen VII plays a key role in anchoring protofibrils at the dermal–epidermal junction. It promotes the ordered assembly of laminin 332 in the basement membrane region, activates its interaction with integrin α6β4, guides the directional migration of keratinocytes, and accelerates the re-epithelialization of the wound surface [57]. It also supports the migration of dermal fibroblasts and regulation of the production of cytokines in skin tissue [58,59]. Therefore, a deficiency of Type VII collagen impairs granulation tissue maturation and prolongs the inflammatory response, resulting in chronic and hard-to-heal wounds [57,60]. During the re-epithelialization of acute wounds, keratinocytes secrete a large amount of transmembrane collagen XVII. As the core transmembrane component of hemidesmosomes, collagen Type XVII not only maintains the mechanical connection between the epidermis and basement membrane by anchoring β4 integrin and laminin [61], but it also dynamically regulates integrin-dependent migration through the PI3K/Rac1 signaling pathway: its intracellular domain directly binds to the PI3K regulatory subunit p85, catalyzing the generation of PIP3 and activating Rac1 GTPase. This induces cytoskeleton recombination (such as the formation of plate-like pseudopodia) and promotes the endocytic cycling of integrin α6β4, thereby coordinating the dynamic balance of “adhesion–dissociation” [62]. This mechanism stabilizes cell polarity at the re-epithelialization frontier and ultimately guides the directional migration of keratinized cells. Conversely, overexpression of collagen Type XVIII impedes wound healing: its C-terminal endostatin domain can inhibit endothelial cell proliferation and migration by suppressing integrin and MMP, ultimately interfering with VEGF-mediated angiogenesis and slowing skin repair [63,64].

Given collagen’s multiple roles in skin wound healing, various collagen-based biomaterials have been modified and processed for skin wound treatment. For example, Qu et al. adopted growth-factor-modified collagen membranes to repair skin wounds. These modified collagen membranes promoted cell proliferation and migration. The wound-healing model indicated improvements in blood flow and epidermal tissue [65]. Recently, Fu et al. [66] prepared an injectable antibacterial collagen hydrogel containing in situ generated silver nanoparticles (AgNPs) for the treatment of diabetic foot ulcers (DFUs). Bacterial infiltration experiments were performed on Col I, Col I/AgNPs-1, and Col I/AgNPs-2 scaffolds in vitro. A large number of bacteria in the Col I group survived and proliferated. In contrast, the numbers of *Escherichia coli* and *Staphylococcus aureus* colonies in the Col I/AgNPs-1 and Col I/AgNPs-2 groups were significantly reduced. This discovery highlights the powerful barrier effect of Col I/AgNPs hydrogel, preventing bacterial infiltration in vitro. Animal experiments were conducted to evaluate the in vivo efficacy of these antibacterial barriers in diabetic wounds by examining bacterial infection in different granulation tissues. By day 7, negligible bacterial presence was observed in the Col I/AgNPs-1 group, while no bacterial colonization occurred in the Col I/AgNPs-2 group. In contrast, the control group still exhibited obvious bacterial infection, whereas the infection rate in the Col I group was only 40%. Wound closure assessments showed that the wound-healing rates in the Col I/AgNPs-1 and Col I/AgNPs-2 groups were higher (>55% on day 4 and >80% on day 7) than those in the control and Col I groups (<30% on day 4 and <60% on day 7). Moreover, wounds in the Col I/AgNPs-2 group were almost completely healed on day 14. These results demonstrate that the Col I/AgNP hydrogel can establish an effective antibacterial barrier; reduce bacterial colonization in wounds; act as a bioactive regenerative scaffold; and promote wound healing, epithelialization, granulation tissue development, and collagen deposition.

### 3.2. Role of Elastin in Wound Healing

EL plays a central role in maintaining skin elasticity [49,67]. Furthermore, EL and its derived peptides activate specific cell-signaling pathways, stimulating various cellular responses [68,69]. This regulatory mechanism plays an essential role in various physiological and pathological processes. EL cannot be produced in sufficient quantities in cases of severe skin injuries [70]. Thus, it is highly advantageous for EL to be introduced into an efficient body delivery system. To this end, Vasconcelos et al. used silk fibroin (SF) in combination with EL in a wound-dressing scaffold to provide the elastin needed for wound repair [71]. Skin burn-healing experiments showed that EL-rich SF/EL scaffolds significantly accelerated re-epithelialization and wound healing. In addition, elastin hydrogels modified with arginine–glycine–aspartic acid (RGD) peptides could promote angiogenesis in vivo [72]. Incorporating EL into a hybrid nanofibrous scaffold of gelatin and cellulose acetate altered the fiber structure and slowed scaffold degradation [73]. EL–collagen composite scaffolds have also been shown to induce moderate EL deposition in rats. In contrast, collagen scaffolds alone did not induce EL synthesis [69]. These findings highlight the importance of biocompatible EL in skin trauma repair.

### 3.3. Role of Laminin in Wound Healing

LM is a significant component of the basement membrane, forming its basic structural framework. The primary LM isoforms in the skin include LM-332 [74], LM-311 [75], and LM-511 [76]. Studies have shown that LM-511 is essential for maintaining adhesion to the epidermis. Additionally, it promotes epidermal–dermal interactions and accelerates skin repair [77,78]. LM plays a multifaceted role in wound healing, promoting wound re-epithelialization and neovascularization, thereby accelerating healing [79].

Several researchers have explored the application of LM-based biomaterials in skin wound repair. Caissie et al. [80] synthesized engineered skin tissue. By adding different doses of LM to the collagen–chitosan (CS) sponge scaffold, the authors evaluated the effect of LM on the regeneration of peripheral nerves. In vivo experiments, in which the scaffold was transplanted onto the backs of mice, showed that compared with the reconstructed skin without LM, the reconstructed skin with 1, 10, and 50 μg of LM exhibited nerve fiber densities that were 2.5, 7, and 4 times higher, respectively (14.1 ± 2.3 fibers/mm^2^ compared with 37.9 ± 11.5, 101.6 ± 23.5, and 62.1 ± 15.8 fibers/mm^2^). In addition, the large number of heparin-binding domains (HBDs) in LM improved the biomaterial matrix performance as a growth factor reservoir and cellular scaffold for effective tissue regeneration [81]. When FN alone proved insufficient to prevent GF loss, the covalent binding of LM HBDs improved GF retention and significantly enhanced the efficacy of growth factors in promoting wound healing (*p* < 0.01).

### 3.4. Role of Fibronectin in Wound Healing

FN is widely present in the ECM and represents the major non-collagenous glycoprotein. FN assembles into a complex network of fibers during cell-driven processes [82]. This network acts as a scaffold, supporting other ECM proteins such as collagen and proteoglycans [83], which facilitate ECM maturation, mechanical signaling, and cell migration. FN is typically classified into two types: plasma and cellular FN [84]. Plasma FN promotes clot formation and coordinates ECM assembly in the early stages of skin damage. In contrast, cellular FN regulates tissue remodeling in the later stages [83].

FN contains a signature RGD sequence that is highly specific for interaction with α5β1, platelet-free αIIbβ3, and αv-like integrins [85]. Within its neighboring structural module, the FN also harbors a functional region known as the “synergistic motif.” This motif binds to the α5β1 and αIIbβ3 integrins (but not αv-like integrins), regulating the formation of capture bonds during binding [86]. The role of the FN synergy site in the healing of the cutaneous wounds of mice carrying dysfunctional FN synergy motifs was investigated by Gimeno-Lluch et al. [82]. Routine wound healing was observed for Fn1syn/syn (mutant) mice. However, in the early stages of wound healing, the wounds in the Fn1syn/syn mice formed less granulation tissue and exhibited reduced myofibroblast content. Vitro experiments revealed that the FN synergistic site is critical for the mechanical tension required for cell migration and GF release from the ECM. In addition, FN isoforms exhibited greater efficacy than full-length FN in selectively promoting wound healing, owing to differences in the availability of binding structural domains [87]. Elevated VEGF, endothelial cell proliferation, and angiogenesis were observed in the presence of extra domain-B fibronectin (EDB-FN) [84]. Currently, EDB-FN is being actively explored to counteract endothelial dysfunction induced by diabetes by promoting cell proliferation [84]. In addition, EDB-FN is involved in immunomodulatory pathways, and EDB-FN synthesis and secretion support the phagocytosis of immune cells targeting inflammatory factors, with potential potentiation in the presence of integrins [88].

### 3.5. Role of Polysaccharides in Wound Healing (Glycosaminoglycans)

Polysaccharides play a crucial role in wound repair owing to their natural, active, and biodegradable properties. Polysaccharides are significantly expressed during skin regeneration and work closely with keratinocytes to promote dermal tissue regeneration. As healing progresses, these polysaccharides gradually fade away, fulfilling their role in promoting skin repair. In recent years, polysaccharides have been increasingly utilized in biomedical applications, including wound dressings and gene and drug delivery systems. Among the various polysaccharides, we discuss GAG, an abundant component of the skin’s ECM microenvironment.

As a critical ECM component [89], GAG forms a gel-like network by interacting with water or other macromolecules. It maintains the biomechanical properties of tissues through the binding of functional proteins [90]. During tissue regeneration, GAGs participate in a positive mechanical–biological feedback loop between the ECM and various cells, transmitting biological signals that regulate cellular responses and promote new tissue formation [91]. Given their diverse roles in maintaining microenvironmental homeostasis, GAGs are considered promising biomaterials for wound-healing applications.

In mammals, six key GAG types have been identified, namely, hyaluronic acid (HA), chondroitin sulfate (CS), dermatan sulfate (DS), keratan sulfate, heparan sulfate, and heparin (HP) [92]. These GAGs play essential roles in the ECM, especially in mature skin, where HA and DS dominate. These GAGs help the skin lock in moisture, thus maintaining its volume and elasticity [93]. When the skin is damaged, the body immediately activates the repair system. GAGs and GAG-based biomaterials promote skin regeneration and repair by modulating the wound microenvironment, accelerating re-epithelialization, and managing ECM remodeling [94]. GAG-based hydrogels demonstrate potential for a wide range of applications in several fields. For example, a heparin-mimetic peptide with antioxidant properties was noted to self-assemble into a hydrogel with a three-dimensional network structure, which significantly accelerated wound healing by inhibiting the initial wound degradation, attenuating inflammatory responses, promoting vascular neogenesis, and optimizing collagen deposition [95]. In addition, GAG is advantageous for microneedle production. Microneedles prepared using GAG are often applied for active ingredient delivery through the skin, aiming to promote tissue regeneration processes. HA is often used to produce soluble microneedles [96]. Typically, GAG-based microspheres are capable of sustained and stable drug release, the protection of active ingredients, and the enhancement of bioavailability at the target site [97]. For example, self-assembled HA microsphere scaffolds, driven by electrostatic interactions, have demonstrated remarkable efficacy in promoting various cellular activities, such as cell proliferation and migration [98]. GAG-containing nanomaterials also typically exhibit beneficial bioactivities. HA nanoparticles, for instance, can effectively induce CD44 receptor clustering and exhibit anti-inflammatory effects [99]. Similarly, antibacterial nanofiber dressings based on CS significantly accelerate burn wound healing owing to their potent antibacterial properties and ability to promote re-epithelialization [100].

### 3.6. Role of ECM-Contained Growth Factors in Wound Healing

The ECM is a known reservoir for various GFs [101]. The ECM also wraps GFs to form concentration gradients that synergistically regulate organismal development and wound repair, revealing a strong dependence between them [102]. When ECM degrades, the GFs bound to it are gradually released, stimulating and regulating cell migration, proliferation, and differentiation behaviors essential for vascularization and tissue regeneration. These GFs impart unique properties to the ECM, rendering it widely used in tissue engineering and repair.

FGF promotes repair during wound healing by stimulating angiogenesis and accelerating cellular activity. Specifically, FGF-2 can drive the development of a neovascularization network, providing essential nutrients and oxygen to the wound [103]. EGF primarily acts on keratinocytes and fibroblasts to stimulate their migratory and proliferative activities, while also contributing to the neovascularization and reconstruction of the epithelial layer. Additionally, it induces the secretion of GF from fibroblasts, further accelerating the wound-healing process [104]. TGF-βs are required for routine wound healing. Animal models of scarless wound healing have revealed perfect scar-free healing of embryonic wounds, associated with the upregulation of TGF-β3 and the downregulation of TGF-β1 and TGF-β2 [105]. VEGF is central in regulating vascular permeability and angiogenesis, promoting collagen deposition and skin regeneration [106]. In addition, during the early stages of wound healing, PDGF acts as a chemoattractant, causing fibroblasts, neutrophils, and monocytes to migrate toward the damaged area, thereby accelerating the inflammatory response and the clean-up process. It also promotes the production of new ECM and induces the differentiation of fibroblasts into myofibroblasts, thereby promoting collagen matrix formation and wound contraction during the proliferation phase [107].

## 4. Decellularized Biomaterials for Skin Wound Healing

Decellularized biomaterials, a significant breakthrough in regenerative medicine, provide an ideal microenvironment for skin wound repair by removing cellular components from tissues to preserve the natural ECM. These materials are derived from human and animal tissues such as the skin, amniotic membrane, peritoneum, and fat [18,108,109]. Their porous network structure can not only promote the migration, proliferation, and differentiation of host cells, but also regulate the inflammatory response and induce angiogenesis by releasing matrix-derived factors, thereby accelerating wound healing [110]. Currently, a variety of decellularized materials have been commercialized and are widely used in clinical practice (Table 2). The following sections summarize studies on the use of decellularized biomaterials for skin wound healing.

### 4.1. Acellular Dermal Matrix (ADM)

**Figure 2:**
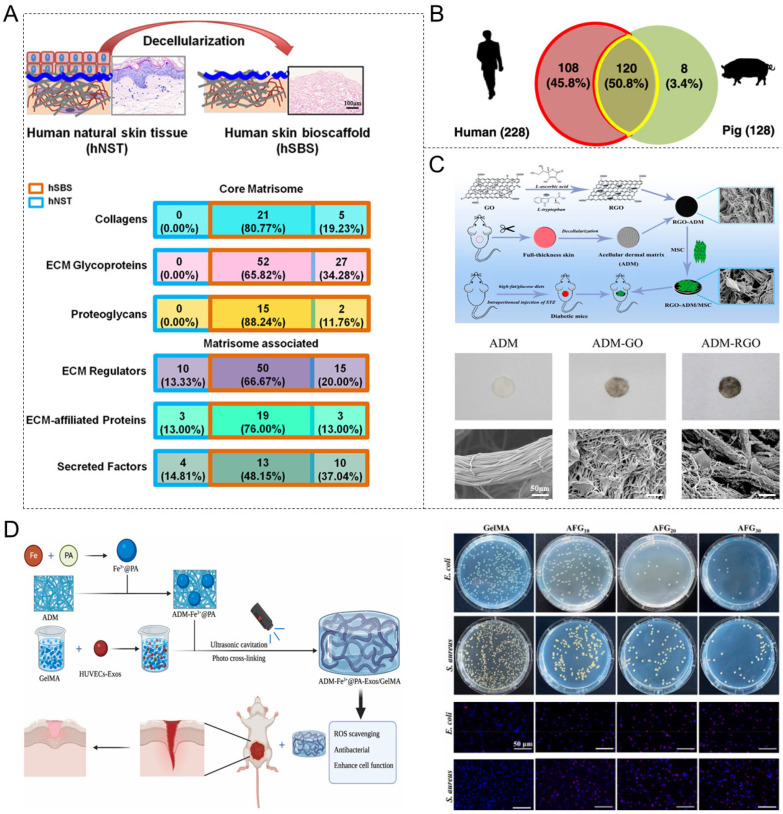
Acellular dermal matrix (ADM) biomaterials for wound healing. (**A**) The construction of a human skin bioscaffold (hSBS) after the decellularization of human natural skin tissue (hNST) and the proportion of classes constituting matrix bodies in hNSTs and hSBSs. Reprinted with permission from Ref. [130]. Copyright 2020 American Chemical Society. (**B**) The number and proportion of matrix body proteins in human and porcine skin bioscaffolds. Reprinted with permission from Ref. [130]. Copyright 2020 American Chemical Society. (**C**) A schematic of synthetic reduced graphene oxide (RGO) combined with ADMs composite scaffolds loaded with MSCs for use in a mouse diabetic wound-healing model, and scanning electron microscope (SEM) images of the three scaffolds. Reprinted with permission from Ref. [137]. Copyright 2019 American Chemical Society. (**D**) The mechanism of ADM-Fe3+@PA-Exos/GelMA promoting diabetic wound repair and images of antimicrobial experiments. Reprinted with permission from Ref. [138]. Copyright 2023 Elsevier. Abbreviation: ADM-Fe3+@PA-Exos/GelMA: ADM modified by trivalent iron ion (Fe^3+^) @ protocatechualdehyde (PA) complex, gelatin methacrylate (GelMA), and exosomes.

ADM is a biomaterial derived from human and animal dermal tissues. Decellularization removes cells and antigenic components while preserving the native ECM [124]. ADM retains key ECM components, such as collagen, FN, and HA, and maintains a naturally porous 3D structure that provides an excellent scaffold for tissue repair (Figure 2A). ADM supports wound healing by regulating, inducing, and promoting host fibroblast proliferation, neovascularization, and epithelialization [125,126].

Depending on the source of the dermis, ADM can be categorized as allogeneic (human-derived) or xenogeneic (animal-derived) (Table 3). Allogeneic ADM, derived from donated human dermal tissue [127], has demonstrated significant efficacy in promoting wound healing in diabetic patients and effectively reducing complication rates [125]. However, its use is limited by the scarcity of donor skin resources and potential risk of disease transmission [128]. Xenogeneic ADMs are primarily derived from porcine dermis, as porcine skin ECM is more similar to human ECM in composition and function than other animal sources [129]. This similarity has been demonstrated by proteomic and bioinformatics analyses of human, porcine, and rat skin scaffold profiles by Liu et al. [130], which revealed high levels of angiogenesis-related proteins in porcine-skin-derived dECM, suggesting its potential in accelerating wound healing by regulating angiogenesis (Figure 2B). Compared with allogeneic ADM, xenogeneic ADM is more readily available and cost-effective for treating skin defects [131]. However, structural differences in major histocompatibility complex molecules can elicit significant inflammatory responses following xenogeneic ADM transplantation (Table 4) [132].

**Table 3:** Comparison of allogeneic and xenogeneic ADMs.

Allogeneic (HADM)	Xenogeneic (e.g., Cattle, Pigs)	Ref.
The collagen amino acid species, quantity, and arrangement are consistent across individuals.	Low homology with humans; a small number of amino acids differ from those in human type I collagen.	[133]
No alpha-Gal antigen and rarely causes immune rejection.	Contains alpha-Gal antigen, which can trigger hyperacute and chronic immune rejection.	[33,134]
1. The pore structure is the same as the human body; 2. Contains human cytokines; 3. Containing human cell recognition signals, with a stronger affinity for human cells, making it more conducive to cell attachment.	1. Pore structure close to human body; 2. Xenogeneic cytokines; 3. Xenogeneic cellular recognition signals.	[133,135]

To mitigate these challenges, methods of using stem-cell technology in conjunction with ADM have been explored. Chu et al. [136] presented ADM scaffolds loaded with human mesenchymal stem cells (MSCs) for diabetic skin-wound healing as a typical example. The MSC-ADM scaffolds significantly promoted the angiogenesis and re-epithelialization of neoplastic skin. They decreased the expression of inflammation-associated factors while increasing the proportion of M2 macrophages in the wound tissue, compared with ADM-alone and MSC-alone groups. To further optimize the therapeutic effect and stem-cell delivery efficiency, Fu et al. [137] combined reduced graphene oxide (RGO) nanoparticles with an ADM (Figure 2C). As a result, they created a favorable environment for stem-cell adhesion and proliferation. In the presence of the nanoparticles, the composite scaffolds supported robust angiogenesis, collagen deposition, and rapid re-epithelialization. In addition to the use of stem-cell technology, Xiang et al. employed a multi-strategy combination approach to enhance the therapeutic efficacy of diabetic wounds. The authors [138] designed a composite hydrogel system that combines ADM modified by Fe3+ @ protocatechualdehyde (PA) complex, gelatin methacrylate (GelMA), and exosomes. This innovative system can promote antioxidant, antimicrobial, and cellular activities and is specifically designed to treat diabetic wounds (Figure 2D).

Current ADM products used for human-skin-wound repair include AlloDerm, GraftJacket, and SureDerm (Table 2). AlloDerm has been approved by the Food and Drug Administration (FDA) through the Class III medical device pre-market approval (PMA) process for the repair of deep burn wounds. It exhibits excellent elasticity, minimizing contractures and scarring. In one study, seven patients with deep burn wounds on different parts of the body surface were transplanted with AlloDerm combined with autologous thin split skin grafts processed from fresh allogeneic skin. Postoperatively, six patients demonstrated favorable graft outcomes, characterized by elasticity, acceptable thickness, and the absence of contracture or scar formation. Graft rejection occurred in only one patient, who had a chronic electrical injury [139]. GraftJacket has also shown a significant therapeutic effect on DFUs, reducing the average healing time by 40–50%. It was approved in the United States through the 510(k) process and has obtained the European Conformity (CE) mark as a Class III device in the European Union.

### 4.2. Acellular Amniotic Membrane (AAM)

The amniotic membrane, the innermost layer of the fetal membranes, has been utilized in trauma treatment since the early 20th century [136] and continues to play a vital role in tissue engineering and regenerative medicine. Its components include several types of collagen (e.g., Types I, IV, V, and VI), FN, LM, and HA [140]. Furthermore, the amniotic membrane is rich in various GFs that play a vital role in promoting wound healing and tissue regeneration [141]. In addition, the amniotic membrane possesses antimicrobial, anti-fibrotic, anti-inflammatory, and low immunogenicity properties [141], representing a valuable natural material for improving wound healing (Table 4) [142].

**Figure 3:**
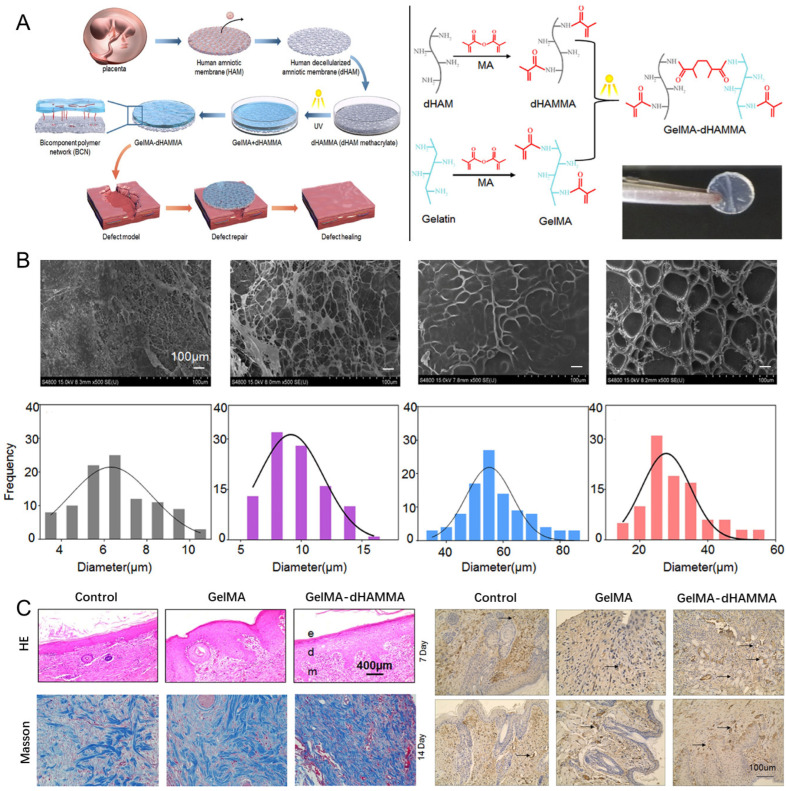
Acellular amniotic membrane (AAM) biomaterials for wound healing. (**A**) A schematic diagram of the preparation of GelMA-dHAMMA BCN skin-defect repair scaffold; the reaction formula of the GelMA-dHAMMA photocross-linked composite and photographs of GelMA-dHAMMA composite hydrogel with BCN structure. Reprinted with permission from Ref. [146]. Copyright 2021 Elsevier. (**B**) SEM images and pore size frequency distribution. SEM images and diameter frequency distribution data plots of dHAM, dHAMMA, GelMA, and GelMA-dHAMMA are shown from left to right. Reprinted with permission from Ref. [146]. Copyright 2021 Elsevier. (**C**) H&E staining, Masson staining, and platelet endothelial cell-adhesion molecule-1 (CD31) immunohistochemical staining of the control, GelMA, and GelMA-dHAMMA groups at the 14th day of trauma. “e” is represented for epidermis, “d” is represented for dermis and “m” is represented for muscular layer. Reprinted with permission from Ref. [146]. Copyright 2021 Elsevier. Abbreviation: dHAM: human decellularized amniotic membrane; dHAMMA: dHAM methacrylate; GelMA-dHAMMA: dHAMMA blended with methacrylated gelatin, BCN: bicomponent network.

For storage, ease of use, and to suppress the immune rejection of amniotic membrane, decellularization is being increasingly applied to prepare AAM. Wilshaw et al. decellularized the amniotic membrane with 0.03% sodium dodecyl sulfate and sterilized it to obtain the AAM [143]. When implanted subcutaneously into the wounds of mice, the AAM promotes the attachment and proliferation of epithelial and stromal cells on its surface. It also facilitates the migration of host cells to the center of the implanted area, thereby promoting tissue remodeling. Similarly, Wilshaw et al. confirmed the cytocompatibility of AAM [143]. In addition, Kshersagar [144] found that, for AAM-treated burn wounds, the AAM contained spindle-shaped migrating cells. The wounds contained well-differentiated repaired epidermis and basement membranes, with prominent hair follicles in the dermis, and exhibited inhibited bacterial growth. These results illustrate the good integration of AAM with the host tissues. Xiao et al. [145] implanted isolated adipose-derived mesenchymal stem cell (AMSC) exosomes on AAM scaffolds and then placed them on the wounds of diabetic animal models. Combined with the AAM scaffolds, the AMSC exosomes regulated inflammatory immune factors at the wound site, promoted peripheral vascular proliferation, and facilitated ECM production to accelerate wound healing. In addition, Zhang et al. [146] decellularized a human amniotic membrane (HAM) derived from the human placenta and grafted it to methacrylic anhydride to form photo-crosslinked HAAM methacrylic acid (dHAMMA). The dHAMMA was mixed with GelMA, inducing photocross-linking to yield a GelMA-dHAMMA composite hydrogel. The GelMA-dHAMMA was found to have a porous structure with a two-component polymer network and favorable physical and chemical properties. In vitro and in vivo studies revealed that the GelMA-dHAMMA promoted fibroblast proliferation and a-smooth muscle actin (a-SMA) expression, facilitated wound collagen deposition and angiogenesis, and supported the healing of large or total skin defects (Figure 3).

AAM scaffolds for skin wound healing include SURFFIXX (CE-marked Class III device), AmnioBand (FDA 510(k)-cleared), Biovance (FDA 510(k)-cleared), and EpiFix (marketed via the HCT/Ps simplified pathway) [120] (Table 2). Because of the inherent properties of the amniotic membrane, AAM exhibits antimicrobial, immunomodulatory, and pain-reducing properties, rendering it suitable for the treatment of a wide range of cutaneous wounds, such as burns of varying severity, pressure sores, or chronic leg ulcers [147]. It has also proven effective in treating thick cleft skin donor sites and chronic DFUs [148]. For example, in a study involving 28 randomized controls, EpiFix (100%) and Amnioband (85%) demonstrated the highest healing rates at 12 weeks [149].

### 4.3. Decellularized Small Intestinal Submucosa (DSIS)

**Figure 4:**
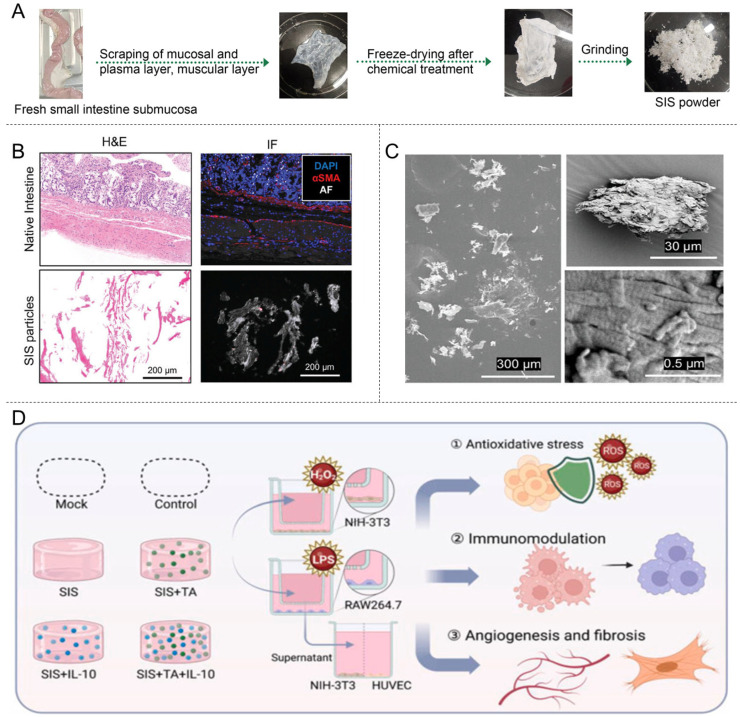
Decellularized small intestinal submucosa (DSIS) for wound healing. (**A**) Decellularization process of porcine SIS. Reprinted with permission from Ref. [159]. Copyright 2023 Elsevier. (**B**) H&E tissue sections of decellularized porcine SIS particles, DAPI, and α-SMA staining. Reprinted with permission from Ref. [160]. Copyright 2024 John Wiley & Sons. (**C**) SEM imaging of the surface morphology of SIS particles. Reprinted with permission from Ref. [160]. Copyright 2024 John Wiley & Sons. (**D**) In vitro functional evaluation of various SIS hydrogels. Reprinted with permission from Ref. [155]. Copyright 2023 Elsevier.

DSIS is a promising biomaterial for promoting tissue regeneration. SIS isolated from the submucosa of animal jejunum is enriched with ECM proteins, including collagen Types I and III, as well as small amounts of collagen Types V and VI. It retains an abundance of GFs, such as TGF, FGF, VEGF, EGF, and FN, which are required for the activation of cell-adhesion signaling pathways and formation of vascular morphology [150]. In addition, DSIS itself exhibits high biodegradability, and during its degradation process, it releases antimicrobial peptides that are inhibitory to both Gram-positive and Gram-negative bacteria. DSIS significantly reduces infections and complications after implantation compared with other graft materials (Table 4) [151]. Therefore, DSIS is a promising candidate material for wound healing, repair, and skin tissue engineering (Figure 4).

Parmaksiz et al. explored the potential of DSIS membranes for trauma repair. They found that in an experimental rat model with a 7 cm^2^ full-thickness skin defect, both the use of DSIS membranes alone and in combination with rat bone-marrow MSCs resulted in complete wound closure within 7 weeks, with no significant difference in closure time between the two groups. In contrast, the wounds in the control group closed by only approximately 47%. This confirmed that DSIS membranes can effectively promote the healing of experimentally induced significant full-thickness skin defects [152]. Additionally, Wang et al. successfully developed a DSIS gel with proangiogenic properties. The gel was noted to promote neovascularization in in vivo experiments compared with collagen Type I (*p* < 0.05) [153]. Kim et al. prepared nanofibrous membranes blended with poly(lactic-co-glycolic acid) (PLGA) and DSIS using single-current electrostatic spinning for improved mechanical properties and biocompatibility [154]. However, the resulting material exhibited insufficient immunomodulatory activity, which limited its application. Zhang et al. [155] developed a novel composite hydrogel material with immunomodulatory properties by innovatively incorporating tannic acid and interleukin-10 (IL-10) into DSIS hydrogel. The composite DSIS hydrogel exhibited optimized swelling and anti-degradation properties, demonstrating excellent free radical scavenging efficacy and sustaining a stable release of IL-10. Owing to its outstanding biocompatibility, ability to promote wound healing, and potential for tissue regeneration, DSIS has been widely used in various clinical treatments. For example, Oasis^®^, a DSIS-derived commercial product, has been approved by the FDA for use in wound healing [156] (Table 2). In patients with chronic leg ulcers, DSIS implantation resulted in higher healing rates at 12 weeks, with healing rates of 40% in the DSIS treatment group compared with 29% in the conventional treatment group [157]. Specifically, DSIS effectively reduced the expression of MMPs and pro-inflammatory factors in chronic wounds (*p* < 0.05) while significantly increasing TGF-β levels (*p* < 0.05) [157]. These findings suggest that DSIS treatment can shift the chronic wound microenvironment toward an acute biochemical state, promoting healing [158].

### 4.4. Decellularized Adipose Tissue (DAT)

**Figure 5:**
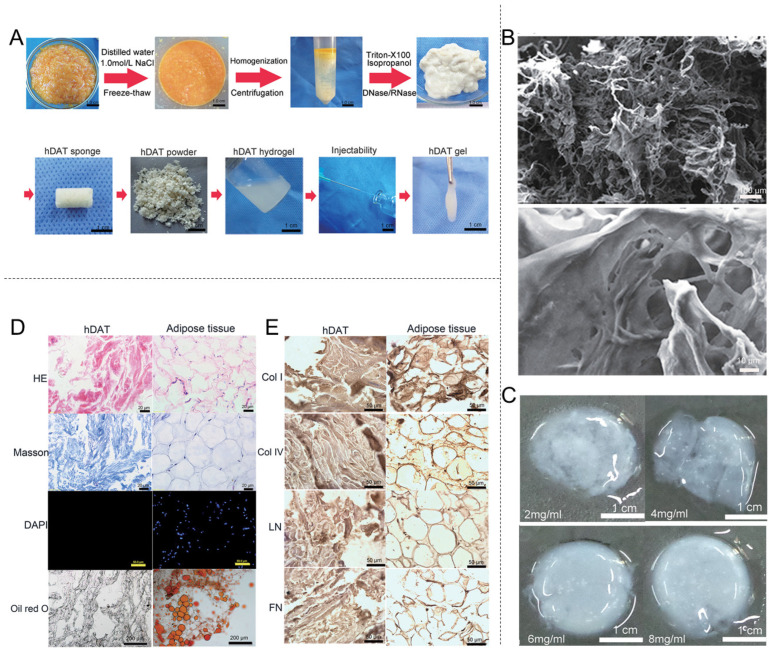
Decellularized adipose tissue (DAT) biomaterials for wound healing. (**A**) Gross morphology of the decellularization process of human adipose tissue and the preparation process of human decellularized adipose tissue (hDAT) hydrogel. Reprinted with permission from Ref. [176]. Copyright 2018 John Wiley & Sons. (**B**) The ultrastructure of hDAT hydrogel under the scanning electron microscope. Reprinted with permission from Ref. [176]. Copyright 2018 John Wiley & Sons. (**C**) The morphology of different concentrations of hDAT hydrogels after gelation for 60 min at 37 °C in vitro. Reprinted with permission from Ref. [176]. Copyright 2018 John Wiley & Sons. (**D**) H&E staining, Masson staining, DAPI staining, and oil red O staining of hDAT and adipose tissue. Reprinted with permission from Ref. [176]. Copyright 2018 John Wiley & Sons. (**E**) The immunohistochemical staining of hDAT and adipose tissue for collagen Type I, collagen Type IV, LM, and FN. Reprinted with permission from Ref. [176]. Copyright 2018 John Wiley & Sons.

Decellularized adipose tissue (DAT) is derived from adipose tissue through a series of processing steps. Human adipose tissue is an excellent source because it is enriched with numerous bioactive ECM components similar to ADM [161,162,163,164,165] and contains stem cells and their precursor cells capable of promoting adipose tissue regeneration and angiogenesis [166,167,168]. Furthermore, DATs lack xenoantigens, such as the α-Gal epitope, minimizing their immunogenicity [169]. The most significant advantage of DATs over other dECMs is their accessibility. Specifically, DAT is typically derived from discarded human adipose tissue from cosmetic surgery and is safely obtained through local anesthesia (Table 4) [170,171]. Following extraction, dECM is washed with distilled water, homogenized, and centrifuged for lipid removal; rewashed; and decellularized to produce dECM (Figure 5).

Recent studies have designed various processes to separate complete ECM from adipose tissue and developed a variety of DAT scaffolds for use in regenerative medicine, such as foams [172], powders [173], microcarriers [174], injectable liquids [175,176,177], and bioinks [178,179]. These scaffolds are especially relevant to patients requiring wound repair. Kim et al. [180] developed a novel hydrogel of DAT and methylcellulose that promoted healing in a whole-skin wound model. Furthermore, Xia designed an injectable DAT scaffold capable of incorporating adipose-derived stem cells (ASCs) to repair skin defects [181]. The experimental data revealed that the DAT-ASC combination exhibited significantly enhanced platelet endothelial CD31 positive expression compared with the control group, underscoring the role of DAT-ASC in promoting the formation of microvascular networks. Woo et al. developed an innovative bilayer dressing combining the advantages of titanium dioxide, chitosan, and DAT for treating normal and infected wounds [182]. The results showed that this dressing significantly inhibited *Escherichia coli* and *Staphylococcus aureus*, reducing bacterial survival by 33.9% and 69.58%, respectively. Compared with the controls, wounds treated with this bilayer dressing showed a significant increase in CD31-positive endothelial cells and microvessel density by the second week post grafting. This demonstrates that the dressing possesses antimicrobial properties and effectively promotes key physiological processes in wound healing [182]. Finally, Yu et al. utilized electrospray technology to inject DAT microcarriers into DAT porous foam [172]. In vivo experimental data showed both adipocytes and vascular function at 8 and 12 weeks, respectively, along with evident collagen rearrangement in the foam. Compared with intact DAT scaffolds, the porous DAT foams elicited more intense angiogenic responses in the host tissue and promoted greater inflammatory cell infiltration; therefore, these porous DAT foams were more suitable for wound healing. DAT commercial products such as Integra^®^ DRT are being extensively investigated [119] (Table 2). This product has obtained regulatory approvals from the US FDA (PMA, 1996), the EU (CE certification in the 1990s), and the Chinese National Medical Products Administration (in the 2010s). Its indications cover burn injuries, chronic wounds, and complex trauma repair. A study was conducted to evaluate the success rate of DRT membrane scalp reconstruction and its feasibility in practice. The results showed that epithelial regeneration occurred in 90% of the 40 patients, with no complications reported. Infection was observed in two cases, while bleeding was noted in three cases. The average defect area was 14.7 ± 10 cm^2^. The healing time was 3.8 ± 2.1 months. These results suggest that the DRT membrane can minimize the need for further surgery, although additional research is required [183].

### 4.5. Decellularized Fish Skin

**Figure 6:**
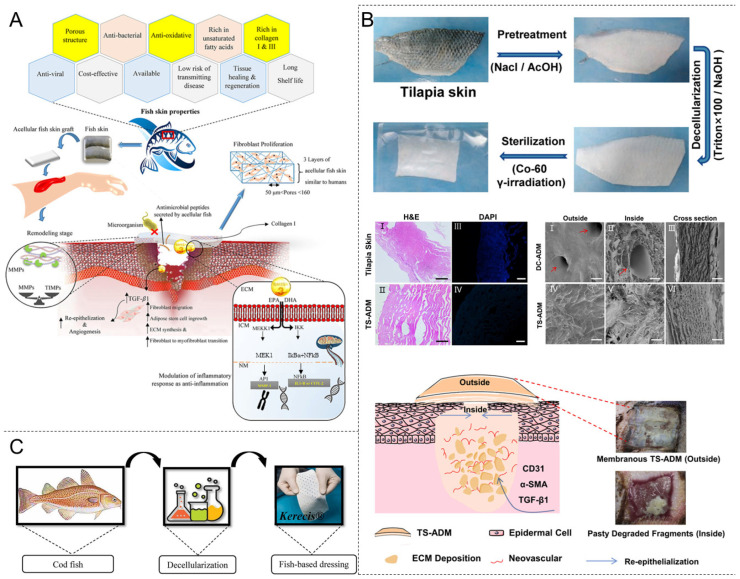
Decellularized fish skin promotes wound healing. (**A**) The effects of decellularized fish-skin properties and Omega-3 polyunsaturated fatty acids on signaling activity during the inflammatory phase of wound healing and mediating tissue remodeling. Reprinted with permission from Ref. [196]. Copyright 2023 John Wiley & Sons. (**B**) Flowchart of TS-ADM fabrication; H&E and DAPI were examined histologically. SEM representative images of DC-ADM and TS-ADM. Among them, the red arrows in Figures I and II indicate the pore structure of the DC-ADM; partially degraded TS-ADM through the formation of a microenvironment favorable for the expression of TGF-β1, α-SMA, and CD31, promoting extracellular matrix deposition, angiogenesis, and re-epithelialization. Reprinted with permission from Ref. [190]. Copyright 2021 Elsevier. (**C**) Kerecis^®^ products represent cell-free Atlantic cod fish skin. Reprinted with permission from Ref. [196]. Copyright 2023 John Wiley & Sons. Abbreviation: TS-ADM: tilapia-skin acellular dermal matrix, DC-ADM: Porcine acellular dermal matrix, TGF-β1: Transforming growth factor-β1, α-SMA: a-smooth muscle actin, CD31: Platelet endothelial cell-adhesion molecule-1.

Interest in decellularized tissues of non-mammalian origin has increased owing to religious restrictions and the risk of zoonotic diseases among mammals. In this regard, denuded marine tissues have been considered due to the similarities that marine and mammalian tissues exhibit in terms of ECM structure and function. Fish skin, a multifunctional tissue, plays various roles, such as chemical and physical protection, sensory activity, behavioral guidance, and hormone metabolism. In addition, as the first line of defense against pathogens, fish skin is constantly challenged by various waterborne microorganisms [184,185]. Fish skin has excellent antimicrobial properties. Mammalian scaffolds must undergo laborious chemical processing to reduce the risk of viral and prion transmission; however, fish are free of α-Gal antigens and have a lower risk of prion diseases and viral infections [186]. Therefore, fish-skin scaffolds are typically subjected to gentler processing methods to better preserve the structure and composition of bioactive substances, including omega-3 unsaturated fatty acids. Omega-3 fatty acids possess antiviral and antimicrobial properties and play a role in modulating inflammation (Table 4) [187,188]. Fish-skin ECM is a promising alternative to mammalian skin due to its delicate physical structure and beneficial bioactive components (Figure 6).

Different fish species have been studied for their ability to repair trauma. Tilapia skin contains large amounts of collagen Types I and III. A tilapia-skin acellular dermal matrix (TS-ADM) mimicking the ECM microenvironment has been noted to promote the healing of acute wounds in rats [189]. Li et al. compared a TS-ADM with a commercial porcine decellularized dermal matrix (DC-ADM) and showed that the former sample was superior in several key performance indices, including morphology, thermal stability, degradability, and permeability [190]. Furthermore, in both rat and minipig skin wound-healing assays, TS-ADM significantly promoted granulation growth, collagen deposition, angiogenesis, and re-epithelialization. The commercialized fish-based ECM product Kerecis Omega3 dressing has obtained FDA certification in the United States. It is derived from the skin of *Gadus morhua* fish and contains omega-3 fatty acids that are beneficial for wound healing (Table 2). When applied to patients with head and neck wounds, it reduced the average healing time from 68 d to 32 d. By the fifth day, the visual analog scale pain score was ≥3 points (*p* < 0.01), and the infection rate decreased from 60% to 0% (*p* < 0.01) [191,192]. Another clinical study compared Suprathel^®^ and Kerecis Omega3 Wound^®^ for the treatment of deep skin burns after enzymatic debridement. Satisfactory outcomes were observed in all 22 cases, with no patient requiring a second debridement after skin grafting. The mean healing time after traumatization with Kerecis Omega3 was 17 d (minimum 8 d, maximum 25 d), whereas the mean healing time in the Suprathel group was 23 d (minimum 12 d, maximum 42 d). No infections were observed in either group. Exudation was significantly higher in the Kerecis Omega3 Wound group than in the Suprathel group throughout the wound-healing process, no significant difference in pain levels was noted. The application of Kerecis Omega3 Wound accelerated wound healing compared with Suprathel and is indicated for the treatment of deep skin burn wounds after enzymatic debridement [193]. The skin of *C. chanos* is rich in biologically active natural compounds, such as collagen. This property, along with the abundance of *C. chanos*, has attracted commercial interest. The decellularization of *C. chanos* skin waste allows for ECM extraction, providing a potential raw material for biomedical applications in skin tissue repair [194]. Furthermore, the skin of many other fish species is considered a high-quality source for collagen extraction and the subsequent production of various biologics.

Manufacturing techniques for various wound-dressing formats have broadened the application of fish-skin dECM. For example, Lin et al. proposed a fish-skin decellularized ECM-based hydrogel textile for wound repair [195]. Fish-derived dECM exhibited ideal biocompatibility, and the textiles prepared using bioprinting technology demonstrated excellent cell adhesion and proliferation performance. The bioprinted dECM hydrogel textiles also possessed tunable porous structures, providing good breathability. Furthermore, the high specific surface area of the porous hydrogel backbone facilitated the loading of various bioactive molecules, further promoting wound healing. In vivo studies showed that these textiles, when loaded with bioactive molecules such as curcumin and basic fibroblast growth factor (bFGF), significantly accelerated chronic wound repair (*p* < 0.01), demonstrating the potential of fish-skin dECM in wound healing and biomedical engineering.

### 4.6. Others

In addition to the several dECMs described earlier, other decellularized tissues, such as decellularized mesothelium, bone matrix, blood vessels, and various organs, have been investigated for treating skin wounds [197,198]. Alizadeh’s team successfully utilized decellularized sheep pericardial tissue to promote skin repair [197]. In addition, Endoform^®^, an ECM membrane derived from the stomach’s antrum, can inhibit matrix metalloproteinase activity and has been widely used in the treatment of acute and chronic wounds, demonstrating remarkable efficacy [123] (Table 2). This product has obtained regulatory approvals from the US FDA 510(k) (as a Class II medical device), the EU CE certification (following the medical device regulations (MDR), Class IIb), and the Australian Therapeutic Goods Administration (in the 2010s), among others.

Urinary bladder matrix (UBM) is used clinically for wound management and soft tissue repair, among other applications. It is primarily composed of Type I collagen and also contains various fibers, basement membrane collagens, glycoproteins, proteoglycans, and ECM-related factors. UBM can effectively shift the persistent chronic inflammatory state of diabetic wounds toward a more acute inflammatory response, thereby reducing inflammation and promoting wound healing. This mechanism is closely related to the effects of UBM on macrophage differentiation and polarization. In vivo and animal model studies have shown that UBM can induce macrophage polarization toward the M2 phenotype, which plays a key role in regulating immune responses, promoting tissue repair, and reducing inflammatory damage, offering a promising approach for diabetic wound treatment (Table 4) [199,200,201].

**Table 4:** The performance of scaffolds from different dECM sources.

dECM	Structure	Immunogenicity	Degradation Rate	Mechanical Modulus	Advantage	Disadvantage
Acellular dermal matrix (ADM) [101,202]	A dense collagen fiber network, preserving the basement membrane structure.	Extremely low (xenogeneic ADM may still contain a small number of α-Gal antigens)	Relatively slow (up to several months after cross-linking)	High (10–30 MPa)	It has wide clinical applications, high mechanical strength, and good biocompatibility, which supports cell infiltration and angiogenesis.	The aperture of ADM is small, and its porosity is low, which is not conducive to cell migration and proliferation, resulting in prolonged healing and scar formation time, and hindering the regeneration of the subcutaneous fat layer; the use of xenogeneic sources may cause mild immune reactions; the source of human materials is limited.
Acellular amniotic membrane (AAM) [203]	Ultra-thin layered structure.	Extremely low (with natural immunity exemption characteristics, with very few residual antigens)	Relatively fast (within a few weeks to 2 months)	Low (0.1–1 MPa)	It expresses the secretion of leukocyte protease inhibitors and elafin, which have anti-inflammatory properties; it produces the antimicrobial peptide β-defensin; its transparency facilitates the observation of the healing process.	Poor mechanical strength; prone to tearing; difficult to use in load-bearing areas.
Decellularized small intestinal submucosa (DSIS) [204,205]	Multi-layer collagen + elastin with preserved growth factors (VEGF, FGF, etc.).	Low	Moderate (2–4 months)	Medium (2–10 MPa)	The antimicrobial peptides produced during the degradation process of DSIS can inhibit both Gram-positive and Gram-negative bacteria. Compared with other grafts, there are fewer infections and complications after implantation.	Limited source (extracted from pig small intestine); uneven thickness; insufficient long-term mechanical support.
Decellularized adipose tissue (DAT) [171]	Loose porous mesh structure.	Extremely low (without α-Gal antigen)	Fast (within a few weeks to 1 month)	Low (0.5–3 MPa)	Easy to obtain; promotes the regeneration of adipose tissue and the reconstruction of blood vessels.	Poor mechanical properties: rapid degradation requires cross-linking; risk of lipid residue.
Decellularized Fish Skin [185,206]	Orderly arranged collagen Type I bundles.	Extremely low (without α-Gal antigen)	Slow (3 to 6 months, and even longer after cross-linking)	Moderately high (5–20 MPa)	It reduces the risk of zoonotic disease spread and circumvents religious restrictions associated with different mammalian species. The Omega-3 unsaturated fatty acids in the fish-skin ECM have antiviral and antibacterial effects and are essential regulators of the inflammatory response.	Clinical data is limited; chemical cross-linking may be necessary to enhance stability; long-term biocompatibility needs to be verified.
Urinary bladder matrix (UBM) [207]	Multilayer composite structure.	Low	Moderate (2–3 months)	Medium (3–15 MPa)	The structure exhibits strong biomimetic properties, making it suitable for urinary system repair, and supports the differentiation of multiple cell lineages.	The preparation is complex, requiring layered processing; mechanical anisotropy may impact the application scenarios.

## 5. Decellularized Matrix Materials for Skin Wound Repair

Skin wounds are classified into two types based on their healing time: acute and chronic. Acute wounds resulting from surgical or traumatic events can be repaired through the routine healing process. Generally, acute wounds are defined as those that heal within the first month from the time of wound formation [208]. However, if any stage of this process is disrupted or delayed, the wound can become chronic, characterized by prolonged or incomplete healing. Delays in wound healing most commonly occur during the inflammatory phase.

The temporal definition of chronic wounds on the body surface has been inconsistently reported in the literature, resulting in no uniform definition. The International Society for Wound Healing defines a chronic wound as one that cannot be repaired in an orderly and timely manner to yield an anatomically and functionally normal state [209]. Clinically, a chronic wound is often defined as a wound that has failed to heal for more than one month and which exhibits no tendency to heal; however, this “one month” is not absolute and can be influenced by factors such as the wound size, wound etiology, and general health of the individual [210]. The treatment of chronic wounds is often tricky, with an extended period being required. There is also a high financial cost. Chronic wounds significantly jeopardize physical and mental health, reduce quality of life, and consume considerable medical resources [211]. Therefore, the treatment of chronic wounds is a key focus in global research. In clinical practice, chronic wound treatment aims to correct the dysfunctional physiological state of the wound, allowing for routine healing to proceed. Like many wound-dressing designs, dECM creates a microenvironment conducive to tissue repair within the wound bed, offering a promising treatment strategy for chronic, non-healing wounds [124,212]. This section summarizes the efficacy of dECM in repairing various types of challenging skin injuries (Table 5).

### 5.1. Burn Wounds

Burns are skin injuries caused by exposure to electricity, chemicals, light, heat, or radiation [217]. The burn wound-healing process is affected by several factors. Burn wounds are categorized as chronic when they do not heal effectively within the expected timeframe and have a recurrent and prolonged course. Globally, approximately 10 million people suffer burn injuries of varying severity each year [218]. Burns are classified by depth as superficial, partial thickness, or full thickness (formerly total epidermal), each with distinct healing outcomes [34]. Treating chronic burns is a major clinical problem and a focus of burn treatment research. Post-burn outcomes are primarily determined by the initial burn wound characteristics, emphasizing the importance of effective burn wound management [219]. While autologous skin grafting is a common clinical approach, it has limitations, including donor site morbidity, limited availability, and the potential for post-operative scar contracture, which can impair function [220]. The development of tissue-engineered skin substitutes, particularly xenogeneic dECM derived from animal tissues, has emerged as a key area of research. Xenogeneic ADM is considered a promising skin substitute due to its potential for rapid vascularization, low immunogenicity, and good stability, making it particularly relevant for treating chronic burn wounds.

Heterogeneous ADM is an almost-cell-free collagen skeleton that can protect burn wounds from bacterial infection, maintain an appropriate local environment, promote the proliferation and differentiation of inflammatory cells and fibroblasts, and accelerate capillary remodeling to accelerate dermal reconstruction [128]. Compared with traditional dressings, applying allogeneic ADM dressings to third-degree burn wounds can reduce the number of dressing changes, wound-healing time, complication incidence, scar proliferation, and degree of proliferation [221]. For third-degree burn wounds, fresher wound granulation tissue and faster wound healing were observed following coverage with xenograft ADM. Further, no obvious pigmentation of the skin and better elasticity were observed after wound healing [222]. ADM can be used as a biological dressing for temporary surface coverage following early scab removal and autologous microparticle skin grafting of extensive burns, as well as a dermal substitute for repairing deep wounds.

Chen et al. [213] examined the potential of porcine ADM for accelerating burn wound healing. Seventy rats with second-degree burns (size: 2 cm) were divided into two groups: one received porcine ADM, while the other group was treated with povidone–iodine cream as a control treatment. Histopathological and biochemical analyses were performed on days 1, 3, 5, 7, 10, 14, and 21 to detect PCNA, K19, integrin-β1, PDGF, EGF, and FGF. The results showed that, compared with the povidone–iodine control group, the ADM group exhibited a higher wound regeneration rate and more effective outcome, with a significant increase in collagen synthesis (*p* < 0.05). Within 21 d after the burn injury, the PCNA, K19, and integrin-β1 levels in the ADM group first increased and then decreased; all levels were stronger than those of the control group. The PDGF, EGF, and FGF levels rose on the third day, peaked on the fifth day, and gradually declined. Furthermore, the expression of the related GFs in the ADM group was significantly enhanced. These results suggest that porcine ADM effectively stimulates collagen synthesis, promotes stem-cell activity, and upregulates the expression of relevant GFs, synergistically accelerating burn wound healing.

### 5.2. Diabetic Wounds

DFUs, chronic skin wounds caused by diabetes, are among the most recognized and difficult-to-treat complications of diabetes. Globally, more than 425 million people are affected by diabetes, and the lifetime risk of developing DFU is as high as 25% [223]. Even with optimal standard care, less than 30% of DFUs heal within 20 weeks, and if left untreated or inadequately managed, they frequently progress to necrosis or require amputation [224].

In the high-sugar environment characteristic of diabetes, blood vessels atrophy, and chronic wounds remain in a persistent inflammatory stage. In addition to impaired blood supply, hypoxia and elevated levels of reactive oxygen species (ROS) are significant contributing factors to diabetic complications. Recent studies have shown that diabetic wounds are characterized by weakly acidic environments with enhanced MMP activity and reduced tissue inhibitor of metalloproteinase (TIMP) activity. This imbalance disrupts the formation and maintenance of an ECM within the wound bed, hindering angiogenesis (blood-vessel regeneration), re-epithelialization, and collagen deposition. These combined factors ultimately contribute to the protracted healing process observed in diabetic wounds [224].

Fetal bovine ADM has been successfully used to treat diabetic wounds [225]. This matrix is characterized by a high content of Type III collagen, a collagen isoform prominent during embryonic development and early wound healing. In a comparative study involving 40 patients with DFUs and 28 patients with venous stasis ulcers, fetal bovine ADM promoted faster healing of both wound types compared to a commercially available material [226]. Cazzell et al. [121] conducted a study on the application of human ADM in the treatment of diabetic foot wounds. In this study, 132 patients were randomly divided into the D-ADM, GJ-ADM, and conventional dressing groups. The ulcer-healing effect was evaluated through a 24-week follow-up observation (at weeks 12, 16, and 24). The results showed that compared with the conventional dressing group, the D-ADM group demonstrated a significant therapeutic advantage after a single treatment (*p* < 0.05). During weeks 16 and 24, the wound-healing rate of all patients treated with D-ADM was significantly higher than those receiving conventional care (*p* < 0.01). At these time points, the cure rate of GJ-ADM was not considerably higher than that of traditional treatment.

Decellularized matrix products commonly used for treating diabetic wounds include DSIS [227] and UBM [207,228]. These materials retain endogenous VEGF, TGF-β, and other bioactive components during processing, which are expected to accelerate blood-vessel formation and promote tissue regeneration [229]. Chen et al. [214] designed an innovative composite hydrogel (SISAM@HN) utilizing DSIS to expedite the healing of chronic diabetic wounds. This hydrogel exhibited both biological activity and tissue adhesion properties. In vitro experiments showed that SISAM@HN could significantly accelerate cell proliferation compared with the control group (*p* < 0.01). In vivo, a diabetic mouse wound model was established to evaluate the tissue regeneration function of the hydrogel. The results after the treatment period showed that compared with the control group (wound area of 0.221 ± 0.067 cm^2^), the SISAM@HN group (0.041 ± 0.041 cm^2^) promoted wound healing to a greater extent. In addition, histological analysis revealed that the synthetic hydrogel facilitated collagen deposition, reduced inflammation, promoted vascular growth, and demonstrated promising potential in accelerating the healing of chronic diabetic wounds. A key advantage of UBM is preserving an intact basement membrane, which can anchor epithelial tissue to the underlying ECM and contribute to wound healing [230]. Previous studies have shown that combining UBM with standard topical wound care significantly improved healing outcomes in human diabetic wounds and reduced recurrence rates compared with topical care alone [215].

### 5.3. Infected Wounds

Losing skin integrity readily predisposes individuals to microbial infections, and wound infections remain a significant challenge in trauma care [231]. Wound infection is a complex pathological process affecting the functions of inflammatory cells, such as granulocytes and macrophages, potentially disrupting the balance of the inflammatory response. This imbalance can inhibit the migration of cells crucial for tissue repair to the wound site. Persistent infection can further impair physiological function through the activation of numerous inflammation-related factors, insufficient angiogenesis, excessive production of ROS, peripheral neuropathy, and loss of hair follicles [232]. Furthermore, bacterial growth often leads to biofilm formation, which encapsulates the bacteria and hinders the penetration of antimicrobial drugs. Debridement alone cannot remove the bacteria and biofilm; hence, infection recurrence occurs and wound healing is prolonged [233]. Bacterial biofilms represent a significant contributor to chronic skin wounds and a primary reason why these wounds are challenging to manage using conventional methods. Studies indicate that 75% to 90% of chronic wounds harbor bacterial biofilms [234]. While antibiotic treatment can inhibit bacterial growth and compensate for incomplete debridement, prolonged use can promote antibiotic resistance, further complicating the management of infected wounds. Common drug-resistant bacteria encountered in clinical practice include *S. aureus*, *E. coli*, *Acinetobacter baumannii*, and *Pseudomonas aeruginosa* [235]. The emergence of new types of infected wounds and the increasing prevalence of rare and uncommon cases highlight the urgent need for novel antimicrobial dressings to accelerate wound healing.

As an emerging material, dECM has garnered significant attention from both the academic and industrial communities for application in biomedical fields, including wound dressings, surface coatings, and electronic skin. dECM also possesses specific antibacterial activities. For instance, Sarikaya et al. demonstrated that ECM scaffold components derived from the submucosa of the small intestine and the bladder exhibited antibacterial activity. This antibacterial activity seemed to be effective against both Gram-positive and Gram-negative bacteria [205]. Additionally, research has shown that the amniotic membrane and placenta are key sources of natural antibacterial agents in the full-term uterus [203]. Furthermore, the Omega-3 unsaturated fatty acids in fish-skin ECM have antiviral and antibacterial effects and are essential regulators of the inflammatory response [206]. However, during the preparation of dECM, the removal of cellular components may damage the structure and composition of the matrix, affecting components or structures that might have originally had potential antibacterial effects, resulting in a weakened antibacterial effect of dECM. Therefore, dECM is often enhanced through combination or cross-linking with other materials or components that possess antibacterial properties. Xu et al. [236] prepared a novel dECM/Gel/CS scaffold with excellent antimicrobial properties, appropriate mechanical strength, biocompatibility, and high porosity. The scaffold effectively promoted cell growth, established a solid “growth environment” for cell proliferation, and ensured an appropriate degradation rate during new tissue formation due to its proper elastic modulus and degradable properties. In addition, these scaffolds had good antimicrobial activity and could absorb water and proteins, preventing wound infections and maintaining a balance between moisture and nutrients. Chandika et al. [216] developed a novel biodegradable composite nanofiber poly(ε-caprolactone)/dECM-loaded ultrasonic acid scaffold (PEU). This composite nanofiber scaffold exhibited vigorous antimicrobial activity against a wide range of bacteria, including *S. aureus*, *Staphylococcus epidermidis*, *Streptococcus pyogenes*, and *Keratobacterium acnes*, and against fungal pathogens (e.g., *Candida albicans*). Additionally, the composite nanofiber scaffolds demonstrated biofilm inhibition against *Klebsiella pneumoniae* and *P. aeruginosa*. In vivo macroscopic evaluation of full-thickness excision wounds in mice revealed that wounds treated with a composite nanofiber scaffold exhibited the highest healing rate. The wound area decreased by 42.1% ± 13.8%, 7.2% ± 3.4%, and 1.3% ± 1.0% at 7, 14, and 21 days, respectively, compared with the control group. Histological assessments revealed that the PEU-treated group showed accelerated scar-tissue formation and granulation tissue maturation compared to both the control group and other treatment groups. This study suggests that the composite nanofiber PEU scaffold holds significant potential for treating infected full-thickness excision wounds.

## 6. Summary and Outlook

Wound management remains a significant challenge and burden in healthcare. Despite the progress made in drug and surgical treatments, the clinical outcomes are often unsatisfactory due to the limited availability of autologous skin grafting, especially for patients with large wounds. Therefore, it is crucial to develop effective and reasonably priced skin substitutes. ECM-based strategies promote wound healing by regulating key biological processes, including cell migration, angiogenesis, and tissue remodeling. Acellular materials, with their natural bioactive components and 3D topological structure, offer unique advantages for the innovative design of wound dressings. However, complete skin regeneration has not yet been achieved, mainly due to the dynamic complexity of the wound microenvironment, which involves factors such as immune cell activity, pH balance, oxygen tension, enzyme activity, and the presence of microorganisms.

In recent years, the integration of 3D bioprinting technology with dECM has led to significant breakthroughs. By combining dECM-based bioinks with advanced manufacturing techniques, researchers have successfully constructed biomimetic scaffolds with precise topological features. For instance, 3D-printed skin analogs developed using porcine dermal dECM not only replicate the epidermis–dermis microstructure but also demonstrate dual functionalities in promoting angiogenesis and inhibiting scar formation [12]. In bioink development, the composite system of dECM and synthetic polymers has been shown to have significant therapeutic advantages. The bioink developed by Dutta et al. incorporated alginate, gelatin, and polydopamine nanospheres modified with polyamines to effectively induce the polarization of M2-type macrophages and promote the release of exosomes. Subsequently, these M2-type exosome-enriched components were skillfully encapsulated in a bioink composed of collagen and dECM to construct an intelligent repair system with immunomodulatory functions. With further advancements in 3D printing technology, micro-pyramid dressings fabricated via digital light processing technology can directionally guide cell migration through surface micropatterns while achieving intelligent controlled release of GFs, significantly enhancing re-epithelialization efficiency [237]. Moreover, cutting-edge explorations have expanded into 4D bioprinting, developing environmentally responsive scaffolds through the incorporation of shape-memory materials. Zeng et al. [238] utilized fiber-reinforced shape memory polymers that exhibit enhanced mechanical properties, with a 200% increase in tensile strength and a 96.4% shape-recovery rate. Their swelling mismatch mechanism offers novel insights for load-bearing structural design. The dynamic thermoset hydrogel developed by Liu et al. breaks traditional limitations through body-temperature-triggered shape memory and body-fluid-responsive deformation, enabling multi-dimensional programmable reconstruction of minimally invasive implantable scaffolds [239]. These innovative material design strategies provide crucial references for delayed triggering mechanisms (e.g., humidity/enzyme responsiveness) in dECM bioinks, demonstrating broad prospects in personalized tissue repair and regenerative medicine. In addition, future research could focus on utilizing inverse modeling techniques to generate complex tubular structures that mimic natural vasculature through 4D printing, subsequently incorporating them into scaffolds. This approach aims to address the vascularization challenge in skin substitutes [240], which is difficult to achieve with traditional 3D printing techniques.

With the collaborative innovation of manufacturing technology and biomaterials, as well as the driving force of clinical needs, the diversified development path of dECM has gradually become more defined. Currently, dECMs with significant clinical transformation potential include five categories: (1) ADM, especially HADM, which has low immunogenicity and has been fully verified in breast reconstruction; (2) DSIS, which has gradually expanded to soft tissue repair and cardiovascular fields; (3) acellular cartilage matrix, due to its morphological adaptability, has become an ideal scaffold for cartilage defect repair; (4) acellular bone matrix, due to its osteogenic induction ability, has demonstrated outstanding performance in bone regeneration; (5) acellular corneal stroma, providing a new option for corneal repair.

Looking to the future, the deep integration of dECM with advanced manufacturing technologies will drive wound repair into the era of precision medicine. However, to achieve large-scale clinical application, multiple technical and management challenges still need to be overcome. First, owing to the lack of a standardized manufacturing process system and infrastructure, dECM exhibits batch-to-batch variations, resulting in high production costs and lengthy processing. Second, the current regulatory system is disconnected from technological innovation, which not only prolongs the approval cycle but also increases the R&D costs for enterprises. Moreover, the agents used in the traditional decellularization process may introduce toxicity risks, and the lack of long-term biological safety evidence remains a key bottleneck for clinical transformation.

Future development should focus on constructing an innovative system for the precision manufacturing of dECM. a. Optimize decellularization methods to enable effective product design, enhancing decellularization efficiency while preserving critical bioactive components. b. Establish standardized preparation processes by leveraging machine learning algorithms to automatically adjust processing parameters. c. Implement a modular certification system to enforce graded approval processes for key technical stages, including material preparation, bioink formulation, and 3D printing techniques. d. Develop a global dECM implant registry system prioritizing the monitoring of implant-associated risks. Through interdisciplinary collaboration among materials science, bioengineering, and clinical medicine, dECM-based biomaterials are poised to overcome traditional limitations, ultimately enabling precise and personalized tissue repair and organ regeneration, thereby accelerating their clinical translation and application.

## Figures and Tables

**Table 1:** The main components and general functions of skin ECM.

Components	Percentages	Major Function	Ref.
Collagen(s)	50–90	Synthesized by fibroblasts, it regulates cell migration and promotes re-epithelialization.	[49]
Elastin	0.6–7.9	Maintains skin elasticity.	[49]
Laminin	<1.0	It helps maintain tissue structure and promotes cell adhesion and differentiation, enabling cells to adhere to the basement membrane.	[50]
Fibronectin	<1.0	It participates in wound healing, including platelet diffusion and the migration of white blood cells to injured tissues. It helps to promote elastin deposition and the mechanical strength of ECM.	[46]
Glycosaminoglycan(s)	<1.0	A gel-like network maintains the biomechanical properties of the tissue by binding functional proteins.	[51]
Others	<1.0	Including growth factors, etc., to stimulate and regulate the cell migration, proliferation, and differentiation behaviors necessary for angiogenesis and tissue regeneration.	[52]

**Table 2:** Biomaterials and available brands indicated in full-thickness wounds.

Decellularized Materials	Product	Application	Ref.
Acellular dermal matrix (ADM)	DermACELL AWM^®^ LifeNet Health, Virginia Beach, Virginia, USA	Normal and chronic wounds	[111]
	GRAFTJACKET^®^RTM Stryker, Memphis, Tennessee, USA	Normal and chronic wounds	[112]
	Integra^®^ Integra LifeSciences, Princeton, New Jersey, USA	Normal and chronic wounds	[113,114,115]
	Alloderm^®^ Allergan Aesthetics, Branchburg, New Jersey, USA	Normal and chronic wounds	[116,117]
	Dermagraft^®^ Organogenesis, Canton, Massachusetts, USA	Diabetic foot ulcers	[118]
	PermaDerm™ Regenicin, Madison, Wisconsin, USA	Burn wounds	[117]
Decellularized adipose tissue (DAT)	Integra^®^ DRT Integra LifeSciences, Princeton, New Jersey, USA	Normal and chronic wounds	[119]
Decellularized fish skin	Kerecis^®^ Omega3 MicroGraft™ Kerecis, Isafjordur, Iceland	Normal and diabetic	[117]
Acellular amniotic membrane (AAM)	SURFFIXX^®^ NorthStar Medical Radioisotopes/Tissue Regenix Group, Madison, Wisconsin, USA	Normal, chronic and infectious wounds	[120]
	AmnioBand^®^ Musculoskeletal Transplant Foundation (MTF Biologics), Edison, New Jersey, USA	Normal, chronic and infectious wounds	[120]
	EpiFix^®^ MiMedx Group, Inc., Marietta, Georgia, USA	Normal, chronic and infectious wounds	[120]
	Biovance^®^ Acelity/Integra LifeSciences, San Diego, California, USA	Normal, chronic and infectious wounds	[120]
Decellularized small intestine submucosa (DSIS)	OASIS^®^ Wound Matrix Smith & Nephew, Fort Worth, Texas, USA	Diabetic foot ulcers	[121]
Acellular fetal bovine dermis	PriMatrix^®^ Integra LifeSciences, Princeton, New Jersey, USA	Diabetic	[117,122]
Decellularized forestomach matrix	Endoform^®^ Aroa Biosurgery, Auckland, New Zealand	Normal and chronic wounds	[123]

**Table 5:** Application of acellular materials in different skin wounds.

Wound Type	Decellularized Materials	Author	Method	Results	Conclusion
Burn wound	Acellular dermal matrix (ADM)	Chen et al. [213]	Seventy healthy Wistar rats were inflicted with a 2 cm second-degree burn and divided into two groups; one group was treated with porcine ADM and the other with povidone–iodine cream. Biopsies were taken on day 1, 3, 5, 7, 10, 14, 21 for histopathological and biochemical analysis to test PCNA, K19, integrin-beta 1, PDGF, EGF, and FGF.	The results revealed relatively better and faster regeneration after the treatment of porcine ADM, along with a significantly increased synthesis of collagen in the experimental group. PCNA, K19, and integrin-beta 1 showed an increase and then tapered off, and were stronger in the experimental group than in the control group during the 21 days after burns. PDGF, EGF, and FGF levels increased on day 3, peaked on day 5, and then started to decrease, while a significantly enhanced expression of relevant growth factors was observed in the experimental group.	Porcine ADM stimulates collagen synthesis, stem-cell proliferation and differentiation, and expression of related growth factors, ultimately promoting burn wound healing.
Diabetic wound	Acellular dermal matrix (ADM)	S. Cazzell et al. [121]	Diabetic foot ulcer subjects. A total of 53 subjects in the D-ADM arm, 56 in the conventional care arm, and 23 in the GJ-ADM arm (2:2:1 ratio). Subjects were followed through 24 weeks with major endpoints at weeks 12, 16, and 24.	Single application D-ADM subjects showed significantly greater wound-closure rates than conventional care at all three endpoints while all applications D-ADM displayed a significantly higher healing rate than conventional care at week 16 and week 24. GJ-ADM did not show a significantly greater healing rate over conventional care at any of these time points.	D-ADM demonstrated significantly greater wound healing, larger wound area reduction, and a better capability of keeping healed wounds closed than conventional care in the treatment of chronic DFUs.
Diabetic wound	Decellularized small intestine submucosa (DSIS)	Chen et al. [214]	The diabetes model was established using C57BL/6 mice, and circular full-thickness skin wounds with a diameter of 10 mm were created on the back skin of the model mice. The diabetic mice were divided into three groups (n = 15): (1) control (PBS); (2) rhbFGF; and (3) SISAM@HN. The cutaneous wounds were photographed and measured at 0, 3, 7, and 14 days.	SISAM@HN is known to promote cell proliferation, aid collagen deposition, reduce inflammation, and stimulate blood-vessel growth.	The SIS acellular matrix-containing HA hydrogel was able to adhere to wound sites, promote cell proliferation, and facilitate angiogenesis, making it a promising biomaterial for wound dressing in the clinical therapy of diabetic skin defects.
Diabetic wound	Urinary bladder matrix (UBM)	M. Martinson et al. [215]	Medicare claims data from 2011 to 2014 were used to identify beneficiaries with diabetes and foot ulcers. Patients treated with one of four types of skin substitute (Apligraf, Dermagraft, OASIS, and MatriStem) were identified. The skin substitutes were compared in terms of episode length, amputation rate, skin substitute utilization, and skin substitute costs.	The percentage of DFUs that healed at 90 days was UBM 62%; SIS 63%; HML 58%; and HSL 58%. Over the entire time, UBM was non-inferior to SIS (*p* < 0.001), and both were significantly better than HML or HSL (*p* < 0.005 in all four tests). Medicare reimbursements for skin substitutes per DFU episode for UBM and SIS appeared to be equivalent. Both were less than HML or HSL (*p* < 0.0005 in all four tests).	UBM and SIS were associated with both shorter DFU episode lengths and lower payer reimbursements compared to HML and HSL, while HML was less costly than HSL but equivalent in terms of healing.
Infective wound	Decellularized extracellular matrix (dECM)	P. Chandika et al. [216]	Establish a diabetic mouse model using ICR mice—full-thickness 2.25 cm^2^ square excision wounds. The mice were divided into control, PCL, PCLU, PE4:6, and PEU4:6 groups and were treated accordingly over 21 days.	PEU 4:6 nanofibrous scaffold enhanced re-epithelialization, dermal tissue maturation, and complete wound closure.	Composite nanofiber PEU scaffolds have great potential in treating infected total wounds.

Abbreviations: PCNA: proliferating cell nuclear antigen, K19: keratin 19, D-ADM: human acellular dermal matrix (ADM), GJ-ADM: active comparator human ADM arm, SISAM: small intestinal submucosa acellular matrix, HN: o-nitrobenzene (NB) was modified onto an HA molecule chain to obtain a phototriggered macromolecule HANB (HN), rhbFGF: Recombinant human basic fibroblast growth factor gel; HA: Hyaluronic acid; DFU: diabetic foot ulcer, HML: Apligraf; HSL: Dermagraft; PEU: composite nanofibrous poly(ε-caprolactone) (PCL)/decellularized extracellular matrix (dECM) scaffolds loaded with usnic acid.

## Data Availability

No new data were created or analyzed in this study. Data sharing is not applicable to this article.

## Glossary

dECM	Decellularized extracellular matrix
ECM	Extracellular matrix
SEM	Scanning electron microscope
H&E	Hematoxylin and eosin
DAPI	4′,6-diamidino-2-phenylindole
SDS-PAGE	Sodium dodecyl sulfate polyacrylamide gel electrophoresis
FDA	Food and Drug Administration
PMA	Pre-market approval
CE	European Conformity
HCT/Ps	Human cells, tissues, and cellular and tissue-based products
NMPA	National Medical Products Administration
MDR	Medical device regulation
TGA	Therapeutic Goods Administration
α-Gal	Alpha-galactosidase
CS	Chitosan
PCL	Polycaprolactone
UA	Usnic acid
ROS	Reactive oxygen species
TIMP	Tissue inhibitor of metalloproteinase
EL	Elastin
LM	Laminin
FN	Fibronectin
EDB-FN	Extra domain-B fibronectin
GAGs	Glycosaminoglycans
MMPs	Matrix metalloproteinases
TSP-1	Thrombospondin-1
DFU	Diabetic foot ulcer
AgNPs	Silver nanoparticles
Col I/AgNP	Collagen–silver nanoparticle
SF	Silk fibroin
RGD	Arginine–glycine–aspartic acid
HBDs	Heparin-binding domains
GF	Growth factor
HA	Hyaluronic acid
CS	Chondroitin sulfate
DS	Dermatan sulfate
KS	Keratan sulfate
HS	Heparan sulfate
HP	Heparin
MHC	Major histocompatibility complex
MSCs	Mesenchymal stem cells
RGO	Reduced graphene oxide
ADM	Acellular dermal matrix
HADM	Human acellular dermal matrix
PA	Protocatechualdehyde
GelMA	Gelatin methacrylate
AAM	Acellular amniotic membrane
AMSC	Adipose-derived mesenchymal stem cells
HAM	Human amniotic membrane
dHAM	Human decellularized amniotic membrane
MA	Methacrylic anhydride
dHAMMA	dHAM methacrylate
a-SMA	a-smooth muscle actin
DSIS	Decellularized small intestinal submucosa
PLGA	Poly(lactic-co-glycolic acid)
IL-10	Interleukin-10
TA	Tannic acid
DAT	Decellularized adipose tissue
ASCs	Adipose-derived stem cells
CD31	Platelet endothelial cell-adhesion molecule-1
TS-ADM	Tilapia-skin acellular dermal matrix
DC-ADM	Porcine acellular dermal matrix
bFGF	Basic fibroblast growth factor
UBM	Urinary bladder matrix
ACM	Acellular cartilage matrix
ABM	Acellular bone matrix
ACS	Acellular corneal stroma
PCNA	Proliferating cell nuclear antigen
K19	Keratin 19
PDGF	Platelet-derived growth factor
EGF	Epidermal growth factor
FGF	Fibroblast growth factor
ROS	Reactive oxygen species
VEGF	Vascular endothelial growth factor
TGF-β	Transforming growth factor-β
